# Early Feminizing Genitoplasty in Girls with Congenital Adrenal Hyperplasia (CAH)—Analysis of Unified Surgical Management

**DOI:** 10.3390/ijerph17113852

**Published:** 2020-05-29

**Authors:** Grzegorz Kudela, Aneta Gawlik, Tomasz Koszutski

**Affiliations:** 1 Department of Pediatric Surgery and Urology, Medical University of Silesia, 40-752 Katowice, Poland; koszut@mp.pl; 2 Department of Pediatrics and Pediatric Endocrinology, Medical University of Silesia, 40-752 Katowice, Poland; agawlik@mp.pl

**Keywords:** disorders/differences of sex development (DSD), congenital adrenal hyperplasia, feminizing surgery, genitoplasty, pediatrics

## Abstract

Aim: To analyze a single-centre experience in feminizing genitoplasty in virilized girls with congenital adrenal hyperplasia (CAH). Methods: Review of medical records of all 46, XX CAH patients undergoing single stage feminizing genitoplasty between 2003 and 2018 was performed. Results: A total of 31 girls aged from 4 months to 10 years were included in the study. The majority (n = 26/31, 84%) were operated before 2 years of age (median 8 months). External virilization was rated as Prader 3 (n = 7/31), Prader 4 (n = 21/31) and Prader 5 (n = 3/31). The urethrovaginal confluence location was low in 19 and high in 12 girls with a percentage distribution similar in Prader 4 and 5 (*p* > 0.05) but significantly different in Prader 3 (*p* = 0.017). The follow-up ranged from 12 months to 15 years. All parents assessed the cosmetic result as satisfactory. Perioperative complications occurred in two patients and included rectal injury (n = 1/31) and prolonged bleeding (n = 1/31). Three patients developed late complications including labial atheromas (n = 2/31) and vaginal stricture requiring surgical dilatation (n = 1/31). Low confluence did not decrease the risk of complications. Conclusions: Early feminizing genitoplasty in girls with congenital adrenal hyperplasia, irrespective of virilization severity, gives satisfactory cosmetic results and is characterized by low and acceptable surgical risk. Nevertheless, the most important determinant of the effectiveness of such management would be future patients’ satisfaction.

## 1. Introduction

Virilization of female genitalia in congenital adrenal hyperplasia (CAH) is a result of enzymatic deficiencies in the adrenal steroidogenesis which increases production of androgens. Deficiency of 21-hydroxylaze is the cause of CAH in over 90% of cases [1]. Female gender identity can be expected in a person with 46,XX karyotype and CAH, therefore early feminizing genitoplasty in these cases seems to be justified. Surgical correction of even very severe virilization in female patients with CAH can restore female-pattern appearance of the genitalia. However some patients’ associations recommend postponing the surgery to the age when the patient can consciously consent for the suggested operation [2,3]. According to the current Endocrine Society guidelines in minimally virilized girls, observation or delayed surgery are preferred, however in severe virilized CAH females, early reconstruction is recommended [4]. A preferable surgical method is an early, single-stage clitoro-vaginolabio-plasty. 

The purpose of the study was to analyze the one-clinical center feminizing genitoplasty management in cases of CAH reared as female with mild to severe virilization (Prader 3–5 rating) and the assessment of early surgical complications.

## 2. Patients and Methods 

The medical records of 46,XX CAH patients operated by single surgeon (GK) in 2003–2018, with 12 months or more follow-up, were retrospectively reviewed. The following data was analyzed: age of operated girls, degree of external virilization, location of urethrovaginal confluence (high vs. low), early and late complications. The analyzed group consisted of 31 girls; 29 girls with 21-hydroxylase deficiency and 2 girls with 11β-hydroxylase deficiency. 

### 2.1. Virilization Assessment and Qualification for Surgery

**Figure 1:**
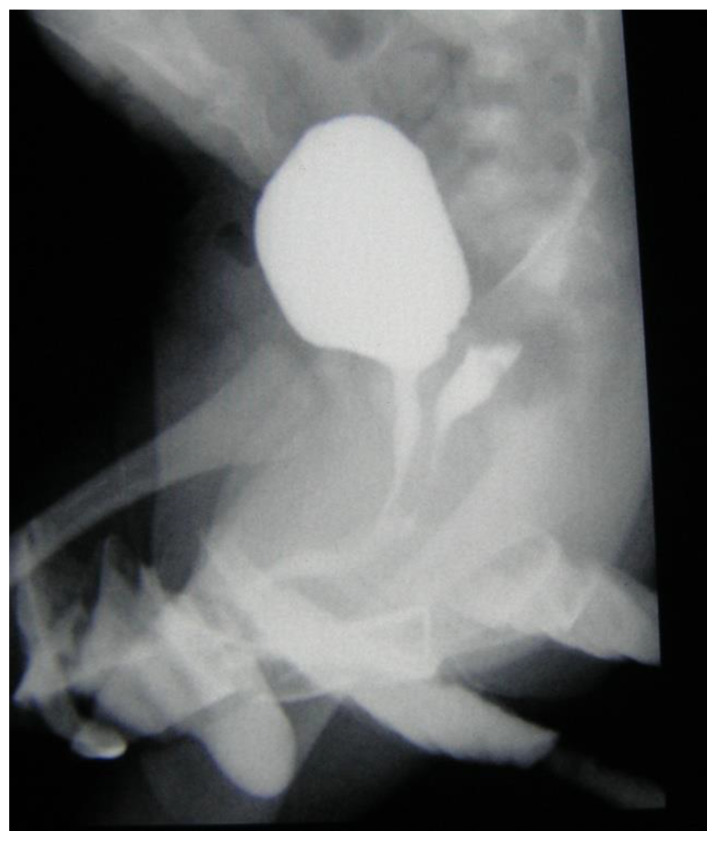
Genitocystography—urogenital sinus with low vaginal confluence.

**Figure 2:**
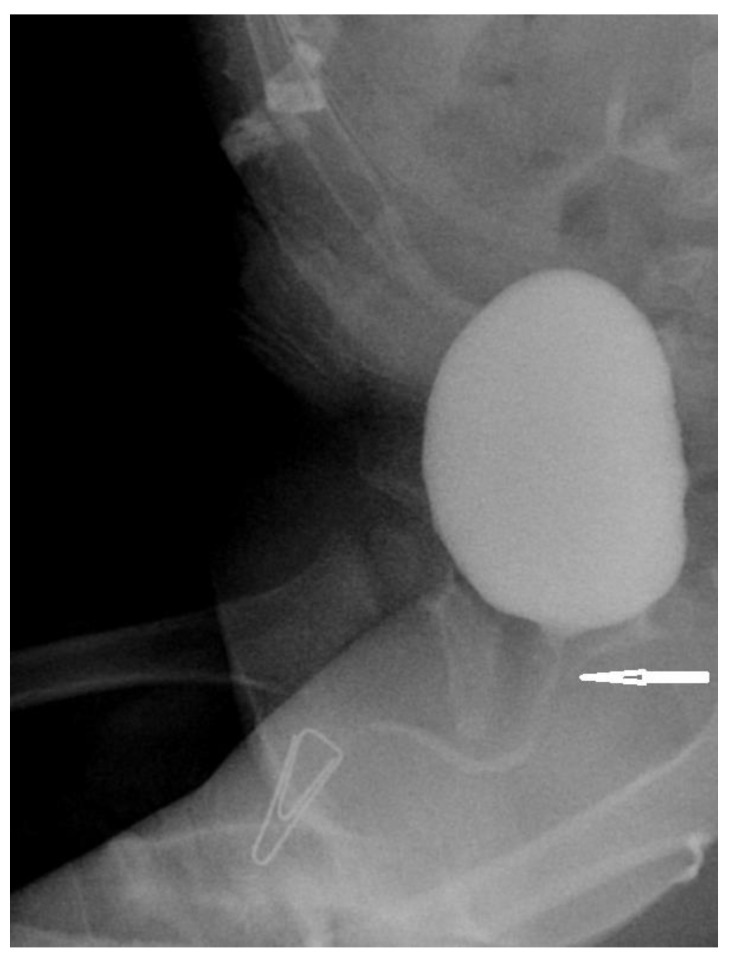
Genitocystography—urogenital sinus with high vaginal confluence. Arrow indicates hardly visible confluence.

The five-stage Prader scale has been used in the assessment of the virilization, however only stages with urogenital sinus (Prader 3–5 rating) were of interest. Stages with separate vaginal and urethral openings (stage 1 and 2) have never been surgically corrected. The urogenital sinus (UGS) has been assessed in genitocystography (Figure 1, Figure 2) or cystoscopy depending on the location of the confluence of the vagina and the urethra: high versus low confluence. As high confluence is often hardly visible (Figure 2) in contrast study, endoscopic study prior to surgery is often necessary. The option of early versus late surgical reconstruction of the genitalia has always been discussed between parents and a multidisciplinary team, including pediatric endocrinologists and pediatric urologists. 

### 2.2. Surgical Procedures

**Figure 3:**
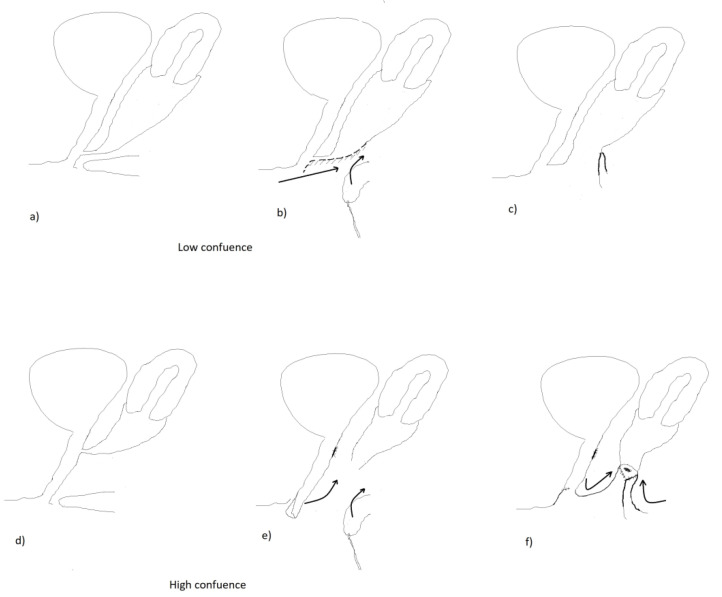
Diagrams showing the separation of the vagina from the urethra in low confluence vs. high confluence malformation. (**a**–**c**) low confluence; (**d**–**f**) high confluence.

A single stage procedure was performed in all patients, however the type of intervention varied according to the level of vaginal confluence. Generally the operation includes the reconstruction of the vaginal orifice and the separation of the vagina from the urethra. In low vaginal confluence malformation, it is performed by a simple U-Fortunoff flap and UGS cut-back. (Figure 3a–c). In higher malformations, the vagina is dissected from the UGS first and then connected with the U-Fortunoff flap. The opening of the UGS after vaginal disconnection is closed with a continuous suture. Distal urethra is then created from the UGS and distal vagina is created from the inverted end of the UGS and U-flap (Figure 3d–f). The clitoris is reduced by dissection of the corpora from glandular tissue and resection at the level of bifurcation. One has to be very cautious to meticulously preserve the neuro-vascular bundle with a strip of tunica albuginea. The enlarged glans is reduced by excising wedges on both sides. Two cutaneous flaps created from the hypertrophic clitoris are sutured to the opened UGS on both sides. Labioscrotal folds are reduced to form a normal-looking labia. Foley catheters are left in the vagina for 2–3 days and in the urethra for 5–6 days.

All data concerning postoperative complications were very thoroughly analyzed. The follow-up visits after the surgery were offered to all patients and were continued both in pediatric urology and pediatric endocrinology clinics. Statistical analyses were performed using STATISTICA 13.1. To compare the fractions Z-test was used. Statistical significance was assumed at *p* < 0.05. The study was conducted in compliance with the Declaration of Helsinki. The Ethics Committee of the Medical University of Silesia approved of the use of retrospective data from patients’ files.

## 3. Results

According to the Prader scale, the external virilization was scored in the analyzed group as follows: grade 3 (n = 7/31), grade 4 (n = 21/31) and grade 5 (n = 3/31). The level of the urethrovaginal confluence was assessed as low in 19 and high in 12 patients. The frequency of high and low confluence was similar in Prader 4 and 5 (*p* > 0.05), and there was significant difference between Prader 3 and Prader 4&5 (*p* = 0.017) (Table 1).

The age of the operated girls was between 4 months and 10 years; mean 19 months, median 8 months. The majority (n = 26/31, 84 %) of children were operated below the age of 2 years (4–20 months). The remaining five patients were operated late: at the age of 5 years (4 girls) and 10 years (1 girl). The reason for delayed surgery was in all cases late presentation in our institution. No one was operated above the legal age of consent which is 16 years in Poland. All parents demanded early genital reconstructions despite the information from the multidisciplinary team about the option of postponing the reconstructive operation until the age of consent.

The follow-up was from 12 months to 15 years (mean 6.8 years). Among intraoperative and early postoperative complications we observed rectal injury in one patient, which was sutured during the same procedure with no further consequence, and bleeding in another patient with subsequent surgical intervention two days after primary operation. A late complication was atheromas growing in the labial area which required surgical removal (two patients). Low confluence did not decrease the risk of complications (Table 2). So far, the surgical dilatation of vaginal orifice was performed in one 17-year-old female patient. The cosmetic appearance after the performed surgery was assessed as satisfactory among all parents. No patient assessed above the age of five years was incontinent and no one has symptomatic urinary tract infections (UTI). Data concerning any sexual activities in our older patients were not collected.

## 4. Discussion

After the consensus meeting in Chicago in 2006 [5] there are still many controversies concerning indications, timing and some technical aspects of surgical treatment in disorders of sex development (DSD) [6]. It has been a topic of wide debate after the 2017 resolution from the Parliamentary Assembly of the Council of Europe, which strongly condemned the medically unnecessary sex-normalizing operations on DSD children [7]. The resolution is only a recommendation for national legislatures, but finally it could become a hard law in any European country.

Patients with CAH and 46,XX karyotype usually have no gender identity problems [8]. The rare exceptions are lately diagnosed severely virilized cases who were assigned at birth and raised as male [9]. Therefore, the majority of parents of 46,XX CAH patients desire early corrective surgery. There is also evidence that classical 46,XX CAH patients do not even wish to be considered as DSD patients [10]. The main aims of surgery in CAH are restoring functional anatomy for future intercourses and reducing risk of urinary tract infection by avoiding fluid (urine and blood) retention in vaginal or uterine cavities. The necessity for corrective surgeries also stems from the parents’ desire to have a girl with female-looking genitalia and spare their child the stigmatization related to atypical genital anatomy. Similar to hypospadias, which is another much more common defect of the genitalia, it is probably better to perform the genital reconstruction between 6 to 18 months of age [11]. This age is believed to be the best for corrective surgery based on psychological aspects [12]. Although there is no clear evidence in the literature showing that early surgery of 46,XX CAH is superior to late surgery, we believe that atypical genitalia including large clitorises may cause much psychological harm to patients and their families. Nevertheless all parents in our institution were informed by the multidisciplinary team that postponing the reconstructive operation until the age of consent could be potentially harmless strictly from the medical point of view. However, all parents did not accept that statement, arguing that protruding clitoris will be socially and psychologically unacceptable. This attitude probably depends on the society’s openness, however it has not changed very much over time. The questionnaire studies in the available literature also indicate that the majority of women with CAH favor early genital reconstruction [13,14]. Additionally most women who were operated on at the older age claim they were operated on too late [15]. The recent psychological study of virilized young women reported three kinds of stigma related to genital atypicalities: experienced, anticipated, and internalized; however, all these various forms of stigma can be reduced by genital reconstructive surgery [16]. The risk of complications of this technically complex operation is considerably low in experienced hands [17,18]. The complications regarding our patients were consequently harmless. None of our patients suffered from urinary incontinence or febrile UTIs. Vaginal stenosis is the most common complication after genital reconstruction [13,16]. Therefore, parents have to be informed about the need of either vaginal dilation and/or introitus surgical correction prior to sexual activity. Our group is still considerably young and only one girl needed surgical dilation of the vaginal introitus.

Meticulous assessment of anatomy before surgery is crucial for achieving good reconstruction results and avoiding severe complications. Defining the level of the confluence between urethra and vagina is of the upmost importance for choosing the right surgical technique. This is assessed by contrast genitocystography or cysto-vaginoscopy [2]. The Prader scale, although adequately describing the severity of virilization, has less practical importance for surgery planning. It is often not correlated to the height of the urethrovaginal confluence [8]. In all our patients with Prader-3, low urethrovaginal confluences were confirmed. Those individuals with Prader-4 and Prader-5 presented both high and low variants, with similar frequencies.

The purpose of the operation is to restore proper functional and female-looking anatomy of the genitalia. This includes separation of the vagina and urethra, formation of the vaginal introitus in the vestibule and clitoroplasty [16]. The technique of the operation differs according to the level of the confluence. The procedure is less complicated in low vaginal confluence malformation, as it includes simple U-Fortunoff flap and UGS cut-back. However, this is not applicable in higher vaginal confluence, in which a more complex technique has to be performed. This includes dissection of the vagina from the UGS and creating its distal part from available tissues: the inverted distal end of the UGS, the cylinder from combined clitoral skin, and the opened USG [19] or autologous buccal mucosa graft [20]. This concept of feminization surgery has been realized in our institution for around the past two decades. We believe satisfactory results of the techniques are dependent on an individual surgeon’s skills and experience. However, according to the literature, the technique involving total mobilization of the entire urogenital sinus and bringing it to the peritoneum [21] can potentially give even less complications [22]. Therefore, total urogenital mobilization is widely recommended nowadays [23]. The neurovascular bundle must be preserved during reduction clitoroplasty regardless of the type of CAH anatomy and performed feminizing genitoplasty [24]. Therefore, we believe that both preserving genital sensation and precise surgical technique focused on good functional and cosmetic result is crucial for future patients’ satisfaction.

It could be suggested that the number of our patients, as well as the lack of the objective surgery sequels, are limitations of this study. However, the findings of our retrospective observation seem important, as they are based on long-term one-clinical center experiences. Nevertheless, we are aware that presented results should be interpreted with caution and may not be generalized to all 46,XX CAH patients undergoing feminizing genitoplasty. Moreover, future study with long-term follow-ups and post-pubertal evaluations together with patient satisfaction with surgery outcomes is highly required.

## 5. Conclusions

Single stage feminizing genitoplasty gives satisfactory results with low surgical risk regardless of the patient’s age at the time of the surgery. In order to destigmatize virilized CAH patients and lessen the psychological burden on them and their families, early surgery can be successfully performed at a young age. Parents’ participation in shared decision concerning therapeutic options should be essential. However, the procedure has to be done in an experienced multidisciplinary center where satisfactory functional and cosmetic effects can be achieved with low risk of complications.

## Figures and Tables

**Table 1:** The percentage distribution of different Prader stages in two variants of urethrovaginal confluence in study population before the surgery.

Confluence n (%)	Prader Rating	
	Score	n (%)
Low 19 (61)	3°	7 (22.6)
	4°	11 (35.5)
	5°	1 (3.2)
High 12 (39)	4°	10 (32.2)
	5°	2 (6.4)

**Table 2:** Types of complications in two variants of urethrovaginal confluence in study population after the surgery.

Confluence n (%)	Complications	
	Type	n (%)
Low 19 (61)	rectal injury	1 (3.2)
	atheroma	2 (6.4)
	vaginal stricture	1 (3.2)
High 12 (39)	severe bleeding	1 (3.2)
